# A novel dual‐wavelength, Nd:YAG, picosecond‐domain laser safely and effectively removes multicolor tattoos

**DOI:** 10.1002/lsm.22391

**Published:** 2015-07-14

**Authors:** Eric F. Bernstein, Kevin T. Schomacker, Lisa D. Basilavecchio, Jessica M. Plugis, Jayant D. Bhawalkar

**Affiliations:** ^1^ Main Line Center for Laser SurgeryArdmorePennsylvania19003; ^2^ Syneron‐Candela Corporation530 Boston Post RoadWaylandMassachusetts01778

**Keywords:** tattoo, picosecond, pulse‐duration, treatment, laser

## Abstract

**Background and Objectives:**

Although nanosecond‐domain lasers have been the mainstay of laser tattoo removal for decades, recent disruptive innovations in laser design have introduced a new class of commercial Q‐switched lasers that generate picosecond‐domain pulses.

**Study:**

A picosecond‐domain, Nd:YAG laser with a KTP frequency‐doubling crystal was used to treat 31 decorative tattoos in 21 subjects. Safety and effectiveness were determined by blinded evaluation of digital images in this prospective clinical study.

**Results:**

The average clearance overall as evaluated by blinded observers evaluating randomized digital photographs was 79 ± 0.9% (mean ± sem) after an average of 6.5 treatments. Of the 31 tattoos completing treatment, 6 had evidence of mild hyper‐ or hypo‐pigmentation by evaluation of photographs.

**Conclusion:**

The 350 picosecond, 532 nm, and 450 picosecond 1,064 nm Nd:YAG laser is safe and effective for removing decorative tattoos. Lasers Surg. Med. 47:542–548, 2015. © 2015 The Authors. *Lasers in Surgery and Medicine* Published by Wiley Periodicals, Inc.

## INTRODUCTION

Q‐switched lasers, capable of delivering ultra‐short pulses of laser energy in the nanosecond‐domain, have been the optimal devices for selective removal of tattoos from skin, and have been used for this purpose for decades. The theory behind the selective use of laser energy to remove targets within skin, without harming the surrounding non‐target tissue was reported in a seminal 1983 paper by Anderson and Parrish 1. They describe the ability to heat target tissue or pigments with a pulsed‐laser by using a laser wavelength that is preferably absorbed by the target and a pulse‐width equal to, or shorter than, the time it takes for the heat to dissipate appreciably from the target to the surrounding tissue 1. Three main types of Q‐switched lasers are currently available and include the 694 nm ruby 2, 3, 4, 5, 755 nm alexandrite 6, 7, 8, and the 1,064 nm and 532 nm neodymium‐doped, yttrium‐aluminum‐garnet (Nd:YAG) laser incorporating a potassium‐titanyl‐phosphate (KTP) frequency‐doubling crystal 9, 10, 11, 12. These lasers are available with pulse‐durations from approximately 50–100 ns as is typical of Q‐switched alexandrite lasers, to about 20–50 ns pulse‐durations of ruby lasers, down to approximately 5–10 ns pulse‐durations available with Q‐switched Nd:YAG lasers 2. Nanosecond‐domain Q‐switched lasers have been the gold‐standard for tattoo removal for decades, with gradual improvements in laser design enabling higher fluences with large beam‐diameters being introduced over many years 12.

Recent disruptive innovations in laser design have introduced a new class of commercial Q‐switched lasers that generate picosecond‐domain pulses 13, 14. Prototype, research lasers in the picosecond‐domain were available 20 years ago and demonstrated effectiveness at removing tattoos 15. Tattoos are created by injecting pigments intra‐dermally after which the ink particles are aggregated in resident dermal cells such as perivascular fibroblasts, mast cells and macrophages, where pigment is mostly contained within membrane‐bound phagosomes in these cells 16. They describe loosely packed particles ranging from 2 to 400 nm in diameter, forming granules within resident dermal cells ranging in diameter from 0.5 to 40 μm 16. These granules are smaller in tattoos that have been treated by a Q‐switched lasers measuring 0.2–1.0 μm, and this suggests that shorter pulse‐durations would be even more efficacious for previously treated tattoos 16. Modeling has suggested that pulse‐durations ranging from 10 to 100 ps would be optimal to fracture the smallest tattoo particles 17; however, it may be the aggregated particles within resident dermal cells that are the most important targets to guide selection of the optimal pulse‐durations. Shorter laser pulse‐durations result in tattoo fragmentation that is more a result of photoacoustic effects than photothermal effects, and may indeed be more efficient at tattoo removal 15. For these reasons, picosecond‐domain lasers were developed to potentially optimize tattoo removal, over standard nanosecond‐domain Q‐switched lasers. We report here a study demonstrating the safety and efficacy of a picosecond‐domain, Q‐switched, Nd:YAG laser delivering 450 ps pulse‐duration 1,064 nm laser energy, and 350 ps pulse‐duration 532 nm laser light.

## MATERIALS AND METHODS

### Subjects

This is a prospective study of the safety and efficacy of a new picosecond‐domain Nd:YAG laser for the treatment of decorative tattoos. Twenty‐six subjects with 36 tattoos aged 19–55, averaging 32 years of age, were enrolled into the current study site. Five subjects with a total of five tattoos withdrew from the study for logistical reasons unrelated to the tattoo treatments, resulting in 21 subjects with 31 tattoos completing the study protocol. This study was approved by an Institutional Review Board (IRB) (Chesapeake IRB, 6940 Columbia Gateway Drive, Suite 110, Columbia, MD) for treatment of human subjects. Subjects were included if they had decorative tattoos that were previously untreated, and no more than 10 × 10 cm in area. Some subjects had more than one tattoo treated, with one having four treated tattoos, one had three tattoos treated, and five subjects had two tattoos treated, while the remaining 19 had a single tattoo treated. Enrollment was open to males and females ages 18–70 with all Fitzpatrick skin types (I–VI). Eight males and 18 females were enrolled in this study site. One subject had Fitzpatrick skin type I, 6 had skin type II, 19 had skin type III and 1 had skin type IV. No subjects with Fitzpatrick skin types V or VI presented for inclusion into the study.

The subject that had four tattoos treated was a female 27 years old, with Fitzpatrick skin type III, and the subject with three treated tattoos was a 25‐year‐old male with skin type III. Of the five subjects who had two tattoos treated in the study, one was male and four were female, their ages ranged from 22 to 42 and averaged 31 years, and four were Fitzpatrick skin type III, while one had skin type IV. Although the most common tattoo color in this study was black, which was present in all tattoos, green, blue, purple, red, and yellow were also present in some tattoos (Table 1).

**Table 1:** Number of Tattoos Containing Various Tattoo Ink Colors

Color	*n*
Black	31
Green	8
Red	6
Blue	2
Purple	2
Yellow	2

### Laser

A prototype, picosecond‐domain, frequency‐doubled Nd:YAG laser system was used for laser treatments (PicoWay®, Syneron Candela Corp, Wayland, MA). The laser delivered up to 400 mJ pulses at 1,064 nm with a pulse‐duration of 450 ps, and pulses of up to 200 mJ of energy at 532 nm with a pulse‐duration of 350 ps. Laser beam diameters were available from 2 to 10 mm which allows maximal fluences of up to 11 J/cm^2^ for 1,064 nm and 5.5 J/cm^2^ for 532 nm. In the current study, beam diameters of 3–5 mm were utilized. The laser repetition rate was also adjustable from 1 to 10 Hz.

### Laser Treatment

For anesthesia, prior to treatment, tattoos were injected with intradermal 0.5% lidocaine with 1:200,000 epinephrine or 1% plain lidocaine, depending upon concurrent medications, while three subjects elected to forgo injections and were treated without any anesthesia (Table 2). Treatments were all performed through a hydrogel dressing (Vigilon; CR Bard, Inc., Covington, CA) to protect the epidermis and minimize the risk of scarring, as well as to prevent the hazard of aerosolized blood and skin impacting the laser operator throughout the laser treatment. The risk of laser reflection may be increased with the use of a hydrogel dressing so, as with all laser treatments, eye protection is imperative. Loss through the hydrogel dressing was measured as less than 10% at 595 nm, and is likely less with 1,064 nm and could be greater at 532 nm 18; however, a hydrogel dressing may benefit treatment by acting as an index‐matching material in the stratum corneum thus enhancing laser energy penetration.

**Table 2:** Treatment Parameters Are Summarized for Each Laser Treatment, for Both the 1,064 nm and 532 nm Wavelengths

	Tx parameter	Txl	Tx2	Tx3	Tx4	Tx5	Tx6	Tx7
1064 nm	Mean fluence (J/cm^2^) (range)	2.0 (1.4–3.1)	3.1 (2.6–5.3)	4.7 (2.4–5.3)	3.5 (2.3–4.8)	2.3 (2.2–2.4)	2.4 (2.3–2.5)	2.5 (2.5–2.5)
	Spot size range (mm)	4–5	3–4	3–4	3–4	4	4	4
	No. of Txs	31	31	31	30	29	28	21
	Median no. of pulses (range)	105 (8–876)	143 (10–886)	157 (25–1175)	174 (30–1096)	220 (29–1203)	284 (29–842)	280 (43–1198)
532 nm	Mean fluence (J/cm^2^) (range)	0.6 (0.4–0.7)	0.8 (0.5–1.3)	1.2 (0.8–2.1)	1.0 (0.7–1.5)	0.8 (0.7–0.9)	0.8 (0.7–1.0)	1.0 (1.0–1.0)
	Spot size range (mm)	4–5	4–5	3–5	3–5	4	3–4	4
	No. of Txs	6	5	6	6	7	4	2
	Median no. of pulses (range)	10 (2–92)	16 (9–39)	36 (3–85)	64 (3–93)	35 (6–176)	81 (14–112)	30 (20–40)
Anesthesia	Lidocaine Inj.	28	29	27	27	25	25	18
	None	3	2	4	3	5	3	3

Mean fluence and the range of fluences, as well as beam diameter or spot size, number of tattoos treated, number of laser pulses, and anesthetic used are summarized.

The treating physician determined the treatment parameters based upon the subjects' Fitzpatrick skin type, the color and intensity of tattoo ink, as well as the clinical whitening response and sound of tattoo pulses upon laser treatment of various portions of the tattoo. Black, blue, green, and purple inks were treated with the 1,064 nm wavelength, while red and yellow inks were treated with the 532 nm wavelength. All tattoos included black ink, so all were treated with the 1,064 nm wavelength. Eight tattoos had either red or yellow ink and were treated with the 532 nm wavelength, although not on every treatment session due to residual erythema or hypopigmentation in the treatment site. Treatments were administered at intervals selected by the treating physician. In addition to black ink, six tattoos had red ink and two had yellow ink. The treating physician utilized a magnifying, cross‐polarizing headlamp to maximally visualize tattoos (v600, Syris Scientific, Gray, ME), which fit over protective laser goggles. The cross‐polarizing headlamp allows enhanced visualization of even faint tattoo ink that may be difficult to visualize under some lighting conditions.

Treatments were administered at 6–10 week intervals, and patients were treated until clinical clearance or lack of continued improvement as assessed by the treating physician, or a maximum of seven treatments. Treatments were administered with beam‐diameters ranging from 3 to 5 mm, with a 5 mm beam‐diameter being used on the first treatment only. Generally the highest fluence available with the prototype system was administered with each spot size at the 1,064 nm wavelength, although this was not the case with 532 nm as sub‐maximal fluences were often sufficient with the selected spot size. Treatment fluences ranged from 1.4 to 5.3 J/cm^2^ with the 1,064 nm wavelength and from 0.4 to 2.1 J/cm^2^ with the 532 nm wavelength (Table 2). It has been shown that for equal fluences, larger beam diameters result in better clearance using a 1,064 nm, Q‐switched laser to treat tattoos 12, so spot sizes and fluences were adjusted by the treating physician to deliver maximal clearance.

### Blinded Evaluation of Digital Images

The treating physician took digital photographs (D80, Nikon Corporation, Melville, NY) at two fixed focal lengths depending upon the size of the tattoo, using the shortest focal distance fitting the entire tattoo within the frame. All photographs were taken with a cross‐polarized flash (Canfield Scientific, Fairfield, NJ) to limit any surface reflection. Cross‐polarization enhances the view of the tattoo over what is normally seen visually with the naked eye by reducing surface reflection, making them look more visible than they are with the unaided eye. At the completion of the study, two types of evaluation were performed by three blinded, physician reviewers. Photographs were taken before each treatment session, 6–10 weeks following each treatment, and 12 weeks following the final treatment session. The final treatment session was after the tattoos were determined by the treating physician to be either clear or not progressing in removal, or after the 7th treatment session. The pre‐treatment and all post‐treatment photographs were placed in a PowerPoint presentation (Microsoft Corporation, Redmond, WA) in a randomized fashion, and graded on a 10‐point scale (0 = no improvement, 1 = 10%, 2 = 20%, 3 = 30% improvement to 10 = 100% or total clearance) for overall clearance and clearance of each color contained within a given tattoo. If an assessor were to incorrectly identify a baseline photograph, the assessor's evaluation would be given a negative score (i.e.,—a score of 3 would be recorded as a −3).

### Side Effects

Immediately following each treatment session pinpoint bleeding, erythema, edema, crusting, and blistering were evaluated by the treating physician using a 3‐point scale where 0 = absent, 1 = mild, 2 = moderate and 3 = severe forms of each treatment effect listed above. Hypopigmentation, hyperpigmentation, and scarring were evaluated by comparing cross‐polarized, pre‐treatment images to those taken 3 months following the final treatment, by the blinded physician observers. Pigmentary alterations or scarring were rated on a 4‐point scale with: 0 = none, 1 = mild, 2 = moderate, and 3 = severe hyperpigmentation, hypopigmentation, or scarring. Comparison with pre‐treatment images would eliminate the possibility of any pigmentary alterations or scarring that were present prior to treatment being mistaken for a treatment effect.

## RESULTS

### Blinded Evaluation of Digital Images

**Figure 1:**
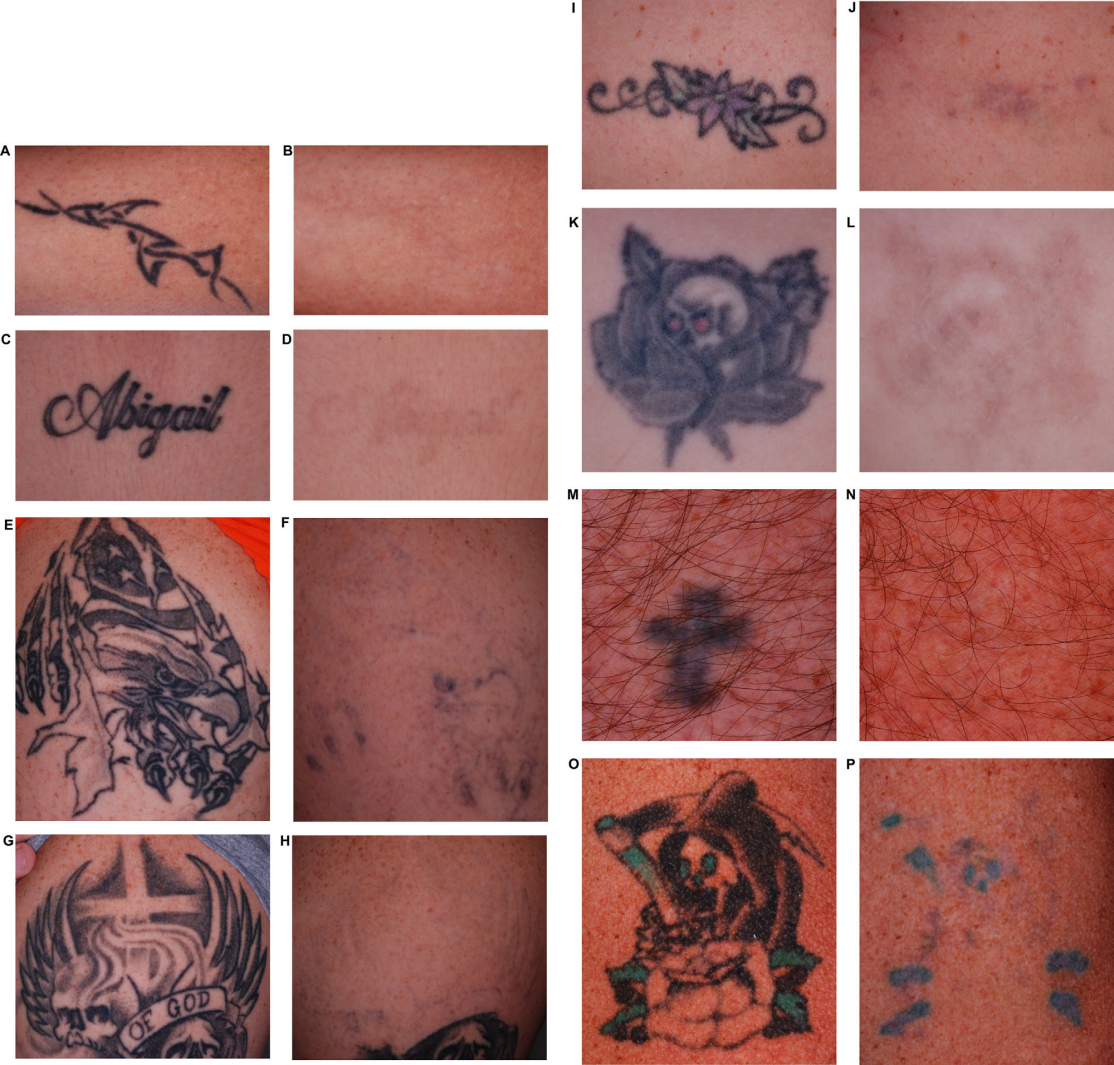
Cross‐polarized, digital images taken before laser treatment (**A**, **C**, **E**, **G**, **I**, **K**, **M**, **O**) and 3 months following the final treatment (**B**, **D**, **F**, **H**, **J**, **L**, **N**, **P**). Black pigment cleared extremely well, while some residual blue (**F**), purple (**J**), and green (**P**) pigment can be seen. Cross‐polarized photography enhances visibility of tattoos over conventional lighting or non‐polarized flash photography.

**Figure 2:**
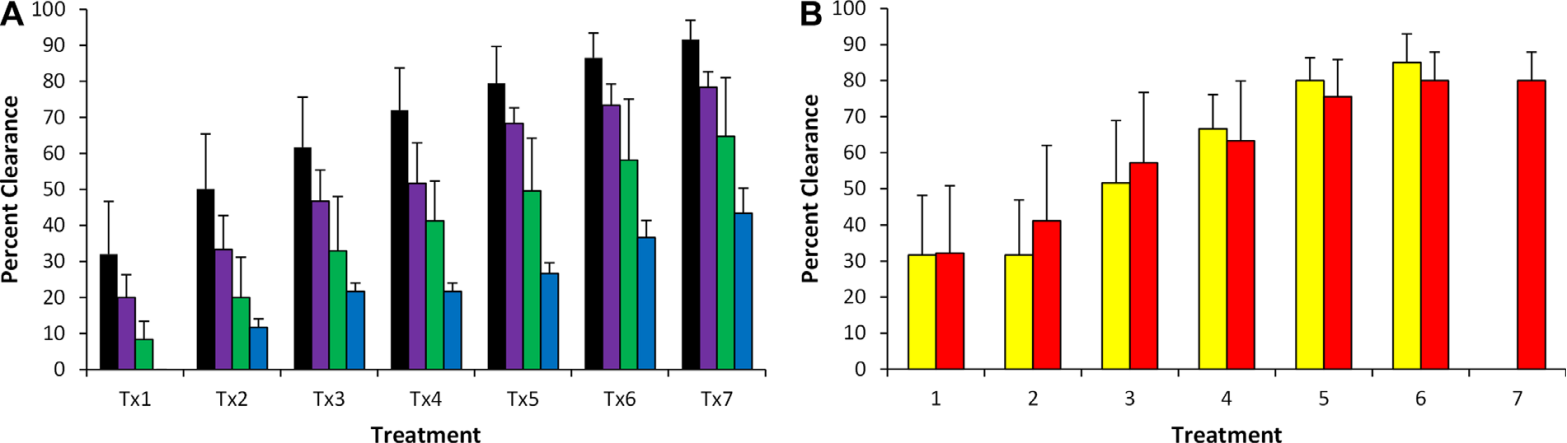
(**A**) Average clearances for each tattoo color treated with the 1,064 nm wavelength are shown following each of seven treatments. Black ink was the most completely removed following each treatment, followed by purple, green, and blue (error bars show sem). (**B**) Average clearances for red and yellow inks following treatment with the 532 nm wavelength are shown for each of seven treatments. Although red ink is typically not difficult to remove with standard Q‐switched lasers, the significant clearance of yellow ink is unusual with conventional nanosecond‐domain lasers (error bars show sem).

Blinded evaluation of digital images by three investigators, blinded as to the treatment conditions, revealed on a 10‐point scale from 0 (least clearance) to 10 (complete clearance) a score of 7.94 ± 0.09 corresponding to 79% removal on average after an average of 6.5 treatments (Fig. 1). For individual colors, clearance scores were 9.16 ± 0.54 (92%), 6.48 ± 0.16 (65%), 7.83 ± 0.44 (78%), 4.33 ± 0.07 (43%), 8.00 ± 0.79 (80%), 8.50 ± 0.80 (85%), for black, green, purple, blue, red, and yellow (Fig. 2).

### Side Effects

Purpura was noted by the treating physician following a single treatment using the 532 nm wavelength and completely resolved by the subsequent treatment visit. Mild pinpoint bleeding was seen immediately following 13.8% of treatments, with associated edema (94.0%) and erythema (69.3%) occurring after most treatments. No blistering was seen immediately following any treatment; however, this could have occurred over the days following treatment and would not have been noted by the treating physician.

Blinded evaluation of photographs, comparing pre‐treatment images to post‐treatment images to rule out any pre‐existing pigmentary alterations or scarring, revealed pigmentary alterations in six tattoos 3 months following the final treatment. Three black tattoos demonstrated mild hyperpigmentation, two tattoos had mild hyperpigmentation in the area of black ink and mild hypopigmentation in the area of red ink, while one tattoo demonstrated mild hypopigmentation within an area where yellow ink was treated. No scarring, or moderate or severe pigmentary alterations were seen in the 3 month follow‐up cross‐polarized images. All areas of hypopigmentation were in portions of a red or yellow tattoo treated with the 532 nm green wavelength, while hyperpigmentation was noted only in black tattoos treated with the 1,064 nm wavelength.

## DISCUSSION

The results of this study demonstrate that this picosecond‐domain, Nd:YAG laser is safe and effective for removing decorative tattoos. As expected, black and red pigment were removed very effectively, with an average 92% clearance of black ink after an average of 6.5 treatments in all 31 treated tattoos, and an average improvement of 80% in the red portions of the six tattoos containing red ink after an average of 4.5 treatments. In this study, only two tattoos contained yellow ink, and while conclusions regarding the ease of removing this normally difficult‐to‐remove color cannot be generalized from two tattoos, the 85% clearance of yellow ink after only an average of 4.0 treatments was both surprising and encouraging. To this point, Geronemus' group recently published a series of six cases of dramatic clearance of yellow tattoo ink using a different 532 nm, picosecond‐domain laser demonstrating greater than 75% clearance in five subjects after 2–4 treatments and complete clearance in one subject after a single treatment 19. Determining the reason for apparent greater clearance of yellow ink using picosecond‐domain versus nanosecond‐domain laser pulses may help optimize laser treatment of all colors in the future. As is the case with nanosecond‐domain, Nd:YAG lasers operating at 1,064 nm, black ink responded the best to laser treatment, while green, blue, and purple ink responded much more slowly, despite often quite dramatic acute responses to laser treatment that often exceeded the acute reactions seen in black ink in the same tattoos. It was hoped that picosecond‐domain lasers would be “color blind” and remove all colors equally; however, we found this not to be the case. The strong response of yellow ink to 532 nm picosecond‐domain may reflect a better match between the picosecond‐domain pulse‐durations and the size of yellow aggregated ink particles. Blue and green inks were less effectively removed by the picosecond‐domain, 1,064 nm wavelength as is also the case with conventional Q‐switched, Nd:YAG lasers. Alexadrite lasers are optimal for removing blue and green pigments, with both nanosecond‐ and picosecond‐domain pulse durations 6, 7, 8, 13, 14.

Laser theory predicts that picosecond‐domain pulses should be more effective than nanosecond‐domain pulses for removing all tattoos, and do so with lower fluences due to the higher peak powers and greater photoacoustic effect over longer pulse‐durations 13, 14, 15, 19. Thermal stress is optimal when the laser pulse‐duration is less than the thermal diffusion time for a particle at the treating wavelength (selective photothermolysis) 1, while acoustic stress is optimal when the laser pulse‐duration is less than the acoustic diffusion time (selective photoacoustolysis) 17. Thermal and acoustic diffusion times refer to the amount of time it takes for a laser target, in this case the tattoo particle, to lose heat or pressure to the surrounding, non‐target skin. If pulse‐durations are too long for a given particle size, too much energy can transfer to the surrounding skin, damaging it and creating a scar.

**Figure 3:**
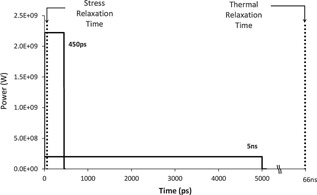
Differences in peak‐powers are easily seen when comparing equal fluences delivered over 5 ns as compared to 450 ps. Diameter of particle is 500 nm.

Tattoo pigments contain nanoparticles, with black inks generally containing the smallest particles which consist mostly of nanoparticles, while white inks are often mixed with various colors to produce softer shades and contain the largest particles, and colored inks are intermediate in size.^20^ Tattoo ink particles are found within phagosomes and free in the cytosol in resident dermal cells such as fibroblasts, mast cells, and macrophages and range in size from 2 to 400 nm in diameter, forming aggregates that can vary in size from 0.5–40 μm 16, 17. Stress diffusion time scales linearly with the particle diameter, while the thermal diffusion time scales with the square of the particle diameter. The smaller the particle diameter, the shorter the pulse‐duration required for optimizing stress on the particle, thus maximizing its destruction. Using computer simulations, Ho et al. calculated that sub‐nanosecond laser pulses are more efficient at rupturing tattoo particles than nanosecond‐domain pulses, due to the increased photoacoustic effect of picosecond‐domain pulses 17. They also calculated that optimal pulse durations for fracturing tattoo particles are in the 10–100 ps range 17. Ross et al. demonstrated as early as 1998 that a 35 ps pulse‐duration Nd:YAG laser was more effective at clearing black tattoos than a 10 ns pulse‐duration Nd:YAG laser in 12 of 16 treated tattoos, using identical settings such as fluence and beam diameter 15. This makes perfect sense, since keeping fluences equal would result in dramatically higher peak powers with the picosecond‐domain device (Fig. 3). In Ross' study, the beam diameters were quite small at 1.4 mm, and this limitation may have hindered the ability to obtain an acceptable clinical outcome for comparison purposes, although their lasers generated extremely impressive output energies and pulse‐durations for a device of its day 15. Ross et al. compared equal fluences and beam‐diameters with the two devices, and thus determined the greater effectiveness of picosecond‐domain pulse‐durations versus nanosecond‐domain pulses of equal energies 15. With the advent of production lasers generating picosecond‐domain laser pulses, relative demonstrations of the ability of these devices to effectively remove tattoos as compared to traditional nanosecond‐domain Q‐switched lasers are needed. This requires reports studying the maximally tolerated fluences (MTFs) that can be delivered with modern devices, by varying fluences and using beam‐diameters that maximize clinical outcomes 12. Comparing identical fluences is not helpful in demonstrating which device is better at removing tattoos, but comparing MTFs is. Our opinion is that beam diameters smaller than a true 3 mm‐diameter spot significantly compromise the clinical outcome of tattoo treatments and potentially increase the risk of side‐effects, due to scattering of laser energy and its relatively superficial deposition in non‐target tissues above the tattoo granules. Thus, the maximum fluence that can be delivered with a 3 mm‐diameter beam may be considered the MTF for a given Q‐switched or sub‐nanosecond‐domain laser.

The true benefits of picosecond‐domain devices for tattoo removal and other applications should become more apparent as these devices are used more frequently in clinical practice. The ability to remove yellow ink more effectively is one quite obvious benefit that presented itself unexpectedly to us, and has been reported by Geronemus' group 19. Both Gernonemus' and Dover and Arndt's groups demonstrated rapid removal of blue and green tattoo pigments using the picosecond‐domain alexandrite laser 13, 14. Geronemus' group has a very large cohort of tattoo patients and concluded that the picosecond‐domain laser is more effective at clearing blue and green pigments than traditional nanosecond‐domain devices 14. In addition to potentially more rapid clearance of tattoos with fewer treatments, the effect of these new devices on faint, incompletely removed tattoos, white or cosmetic tattoos containing iron or zinc oxides, and traditionally difficult‐to‐remove colors should be studied. Picosecond‐domain lasers are already being used for skin rejuvenation and improvement of acne scarring 21, using fractionated and non‐fractionated beam profiles. With more picosecond‐domain devices entering the market, future applications of this technology should expand our already wide‐array of treatment options for a myriad of conditions.
